# The Cold Futures of Mouse Genetics: Modes of Strain Cryopreservation Since the 1970s

**DOI:** 10.1177/01622439221138341

**Published:** 2022-11-15

**Authors:** Dmitriy Myelnikov, Sara Peres

**Affiliations:** 1 Department of History and Philosophy of Science, University of Cambridge, United Kingdom; 2 Geography and Environmental Science, University of Southampton, United Kingdom

**Keywords:** archiving and collecting practices, futures, alternative life forms, cryopolitics, animal research, mouse genetics

## Abstract

Cryopreservation, or the freezing of embryos or sperm, has become a routine part of many research projects involving laboratory mice. In this article, we combine historical and sociological methods to produce a cryopolitical analysis of this less explored aspect of animal research. We provide a longitudinal account of mouse embryo and semen storage and uses in the UK and show that cryopreservation enabled researchers to overcome particular challenges—fears of strain loss, societal disapproval, and genetic drift—in ways which enabled the continued existence of strains and contributed to the scaling up of mouse research since World War II. We use the theoretical lens of cryopolitics to explore three different, yet overlapping, cryopolitical strategies that we identify. All share the ability to ensure the continued maintenance of genetically defined strains without the need for continually breeding colonies of mice. We argue that, in contrast to more common imaginaries of species conservation, the cryopolitical rationale can best be understood as purposefully not letting the strain die *without* requiring animals to live. The ability to freeze mice, then, had the potential to unsettle who the objects of care are in mouse research, from individual animals to the concept of the strain itself.

## Introduction

Archiving is something that we’re really putting in place for future generations. I…see it for three reasons really; we’ve got the archiving as part of our contingency plan, so if we do have a disease outbreak or fire or anything like that we don’t have to go back and start a lot of our lines again. Archiving is also great for pausing the breeding…if you can archive the animals and stop [them] breeding…you’ve not just had six months of animals that…you’ve not known what to do with. And then of course the archive is there so that we’ve got these animals available for the researchers…in generations to come.

Leon, facilities leadership

Freezing mouse embryos and semen, or cryopreservation, is a routine procedure in major facilities for mouse genetics. It enables strains to be stored indefinitely in liquid nitrogen and “recovered” by thawing and implantation into foster mothers. Mouse embryos were made reliably freezable in the 1970s; freezing semen has offered a cheaper alternative since the 1990s, albeit one requiring further breeding once thawed.^1^ As the epigraph suggests, the cryopreservation of mouse strains is framed temporally: it allows pauses, prevents “having to go back and start again” and offers a way to accumulate and disseminate mice while reducing genetic changes through time. Lemke (this volume) offers an understanding of such temporal interventions by suggesting that cryotechnology “extends the present” by keeping options open and offering a degree of reversibility. In mouse research, freezing gametes and embryos into biological stasis allows for extending the present of strains, rather than individual mice. It also makes objects available for accumulation and dissemination: once living cells can be frozen and placed in cryobanks, they amount to a “standing reserve.”

Here, we explore how such reversibility enabled mouse researchers to overcome particular challenges—fears of strain loss, societal disapproval, and genetic drift—in ways that enabled the continued existence of strains and helped scale up mouse research. We draw on the recent interest in cryopolitics (Kowal and Radin 2015; Radin and Kowal 2017; Lemke 2019) to emphasize the normative and sociopolitical significance of cryopreservation. Cryopolitics refers to the political, social, and cultural implications of the intersection of time and low temperatures. It focuses on how freezing practices create “latent” (Radin 2013) or “suspended” (Hoeyer 2017) life in ways that destabilize categories such as personhood or property, as well as binaries between self and other, and indeed life and death (Lemke 2019, 452).

If biopower is expressed in terms of “making live and letting die” (Foucault 2003), cryopower is about “produc[ing] a zone of existence where beings are made to live and are not allowed to die” (Radin and Kowal 2017, 6). One of the major lessons from cryopolitical investigations is that “not letting die” can, paradoxically, be a form of letting die rather than making live (Radin and Kowal 2017, 8). The inverse—the idea that cryopreservation may be desirable because it might “not let die” *without* letting live—is, we argue, central to the cryopolitics of laboratory mice. TallBear (2017) has critiqued cryopreservation practices around biological sampling of indigenous peoples, arguing that the communities from whom samples are collected are not normally seen as potential beneficiaries of the knowledge being sought. The value and promise of indigenous DNA preservation is predicated on narratives of indigenous extinction left unarticulated. In the case of mouse genetics, by contrast, not letting live is a virtue accepted by most stakeholders as a desirable outcome that would bring the gradual reduction of animal experimentation and even its possible end. The preference for not-life documented here is, to our knowledge, unique among studies of cryopreservation to date, but we hope that other empirical cases may emerge.

Cryopreserving the reproductive substances of laboratory animals presents a specific set of concerns. Since mice are laboratory animals, their existence is entangled with that of specific research projects (that have their own timescales and endings). Yet unlike frozen blood, DNA, or somatic tissue samples, embryos and semen are not just research substance or scientific proxies for mice (Parry 2004); through embryonic technology, they enable the recreation of new generations of living organisms and revitalizing the types of animals that had not been alive for decades. By contrast with human medicine, it is not future individuals that are the primary subject of preservation or fertility that is being deferred—nor is it the species at large as in wildlife preservation (Van de Wiel 2021; Friese 2013; Chrulew 2017). Rather, mouse embryos and gametes are frozen to preserve a *strain—*a specific breeding population of mice (Parry 2020). Landing between the levels of the individual animal and species has been productive in studies of breeds (Franklin 2007; Woods 2017). Focusing our analysis on freezing puts into relief the relationship between the ontological concept of the strain, and the individuals that make up populations. Cryopreservation unsettles the objects of care in mouse research—from individual animals to the concept of the strain itself, and it has yielded new ways of attending to mouse research.

In this article, we argue that the government of life on ice is as much about “not letting live” as it is about “not letting die.” Cryopreservation in animal research is aligned with hope and the drive to protect—to safely back up, to circumvent loss—yet its appeal and benefits to mouse research lie in providing a means of preserving strains without continual breeding of mice. Hence, suspending life at an embryonic level is less of an unintended effect of cryopreservation that defers action and deadens hope; it is crucial to the success of contemporary mouse research. Cryopreservation thus enables further instrumentalization of both the laboratory animals and forms of care for their lived experiences.

Empirically, we provide a longitudinal account of mouse embryo and semen storage and explore the different, yet overlapping, cryopolitical “modes” or strategies that we identify in the following sections (see Table 1). The strategies we describe show that while cryopreservation is primarily a means to protect the strain, alternative arrangements have obtained between living populations of animals and their frozen embryos. Each is dominated by specific concerns, in different periods and with different risks and opportunities emphasized. Yet, notably, all are grounded on the assumption that it is preferable to keep strains as frozen embryos and sperm, rather than as living animals. Hence, in the Extending the Present by Keeping Strains on Ice subsection, we discuss what this account contributes to our understanding of cryopolitics.

**Table 1.** Three Modes of Cryopreserving Mouse Models.

Priority	Security	Responsibility	Stability
Prevalent during	1970s–1980s	1990s	2000s
Dominant risk	Vulnerability to accident or limited capacity	Reputational and economic costs of wasting animals	Mutability inherent to animal reproduction
What does the cryobank offer?	Insurance	Efficiency	Quality control
Imaginary of good science	Resilient	Humane/efficient	Reproducible

Methodologically, we bring together contemporary and historical materials. Our archival and published sources and ethnographic work largely focus on the UK. We draw on ethnographic fieldwork (two weeks) and semi-structured interview data (*n* = 23) with participants involved in breeding, supply, and biobanking of laboratory mice in the UK as animal care staff, researchers, and administrators. All participants have been assigned pseudonyms.^2^ Combining historical and sociological modes of investigation allowed for a better engagement with the “intimate entanglement of past, present and future temporalities” enacted by freezing technologies (Lemke 2019, 453), enabling us to consider continuity and change in the practices and discourses of embryo freezing. Historical sources highlighted the narratives of vulnerability, while contemporary interviews and ethnographic observation enabled access to practices and concerns of cryobanks that are difficult to extract from archival or published documents. Combined, these sources foreground the importance of freezing in managing risk, unsettling temporalities, and assuring the future of mouse strains and collections.

### Security: Embryo Freezing and the Survival of Strains

Embryos were the first type of mouse reproductive substance to be reliably frozen since the 1970s, an innovation then welcomed by geneticists eager to reduce costs, preserve little-used strains, and retain the possibility of returning to them in the future. Early discussions of embryo freezing emphasized stories of loss and vulnerability, and the need to return to strains after they had been allowed to die. As Joanna Radin notes, “the Cold War was not only a time of nuclear standoffs between the United States and the Soviet Union. It was an epoch characterized by anxiety about new temporal horizons of risk” (Radin 2017, 6). The concern over losing mouse strains, and the opportunities freezing offered to access more mutants was eagerly embraced by major institutions of mouse supply as well as funders. Mouse genetics and much of early embryo banking were embedded in Cold War institutions of radiation research (Rader 2004; de Chadarevian 2006); preservation, resilience, and protection from the effects of radiation were typically Cold War concerns. In their state of suspended animation, frozen embryos embodied the promise of security through indefinite storage, as well as the potential to recreate life if something were to go wrong—a means to avoid total loss.

A mouse strain is a specific breeding population of mice. In the context of mouse genetics, this is normally an *inbred strain* that is genetically homogeneous,^3^ produced through at least twenty generations of brother–sister crosses. New strains emerge through spontaneous or induced mutation, or genetic modification, but need to be standardized, recorded, and named (Davies 2013a; cf. Bangham 2019). As Bronwyn Parry has recently argued, strains are best understood as collective performative works of multiple stakeholders (Parry 2020). Maintaining strains and their standardization has been a key mission of the major institutions of mouse genetics. In the 1900s, mice were domesticated in the laboratory with the institutionalization of genetics, but it was the mid-century expansion of biomedicine and research into the effects of radiation and cancer that cemented their position among research species (Rader 2004). Like most organism-oriented communities, mouse genetics has relied on the circulation of strains between laboratories and institutions, within a moral economy that prioritized sharing (Kohler 1994). Major centers acted as hubs for supplying and maintaining strains, both nonprofit institutions such as the Jackson Laboratory in the United States or Medical Research Council (MRC) Radiobiology Unit in Harwell, UK, and commercial breeders, all relying on elaborate procedures to assure genetic quality of their standardized mice (Rader 2004).

These early exchanges involved adult animals, but in the 1950s, mouse embryos had started to become mobile, too. Histories of transplanting embryos between animals, or embryo transfer (ET), stretch back to the 1890s, but serious work began after the Second World War across the laboratory and experimental farm, resulting in the first successful births following ET in cows, sheep, pigs, and mice (Betteridge 2003; Hopwood 2018). As new tissue culture media and methods opened up the earliest stages of mammalian development for observation and experimentation, Anne McLaren and Donald Michie at the Royal Veterinary College in London reported ET between mouse strains in 1956 (McLaren and Michie 1956). Their experiments were designed to challenge the assumption that genetic standardization always resulted in physiological uniformity and to explore the effects the uterine environment had on the embryo. From the beginning, issues of storage, preservation, and refrigeration were also at stake, responding to practical concerns of the mouse genetics community. Thus, McLaren considered the possibility of storing and shipping embryos, noting recent successes such as the transatlantic shipment of rabbit embryos refrigerated to +10 °C and packed in a thermos flask (Biggers and McLaren 1958). Making embryos transferrable between organisms also made them spatially mobile.

In exploring transportation and preservation, reproductive scientists concerned themselves with better methods of refrigeration and freezing. In 1949, fowl semen was successfully frozen at the National Institute for Medical Research in Mill Hill, London, and bull semen in 1952 in Cambridge, extending the scope for artificial insemination in agriculture and the distance semen could travel (Radin 2017, 35-45). In 1952, Audrey Smith at Mill Hill demonstrated that rabbit embryos could in principle survive freezing, but it wasn’t until the 1970s that mammalian embryos could be reliably frozen and thawed (Smith 1952; Betteridge 2003). David Whittingham’s experiments in 1971 and 1972, in Cambridge and with cryobiologists at Oak Ridge, TN, revived interest and ambition for cryogenic embryo storage (Whittingham 1971; Whittingham, Leibo, and Mazur 1972). Whittingham, alongside other geneticists and developmental biologists, tinkered with the techniques, testing cryoprotective agents including glycerol and dimethyl sulfoxide (DMSO), as well as freezing and thawing rates.

Early meetings brought key players together and articulated the promises of freezing. At the 1978 Ciba meeting on Freezing Mammalian Embryos in London, Jan Klein, a mouse geneticist from the University of Texas, reflected on what freezing embryos could do for him. When he had moved from Michigan to take up his appointment, 15,000 mice had to accompany him:

I considered several alternatives and found them all impractical, except one. I decided to reduce the number of mice in my colony to about one-half, to load a plane with these remaining mice, and to fly them to Texas…. The plane did not crash; it was not even hijacked; and the mice were soon safely housed in their new quarters. But I quickly realized that my troubles were only beginning, since it was apparent that something was causing a definite slowdown in breeding. Was it the combined effects of transportation stress, adjustment to the new environment, and the reduction in the size of the breeding nucleus? I did not know…. At the end, I had to write off some 25 of my unique and irreplaceable strains. Had embryo-freezing techniques been available at the time, undoubtedly I would not have suffered this great loss. (Klein 1977, 306)

Other stories followed. Klein had tried to secure rare *t*-locus mutants form Columbia University, only to discover most had been culled for the lack of space in the animal house. With another gene he had worked with, there were simply too many mutant strains to maintain. The opinion of fellow mouse geneticists was uniform: as Klein relayed it, “Tell [the embryo-freezing scientists] that we are all impatiently waiting for a practical technique which will ease the pressure on our mouse colonies and are longing for a central embryo-freezing facility in which we could store our stocks” (Klein 1977, 305). Cryopreservation was therefore a technological innovation around the corner that would enable geneticists to overcome current limitations to working with living animals alone.

Such limitations included vulnerability of colonies and waste of effort as key matters of concern. Diseases could spread through colonies rapidly, and major resources went into organizing animal facilities to minimize contagion risks (Kirk 2012). Freezing, thawing, and subsequent ET into a specific-pathogen free surrogate mother avoided most disease risks. Other concerns included fire and natural disasters. The Jackson Lab, for instance, suffered a major fire in 1947 and had to rebuild its collection by appealing to the mouse genetics community (Rader 2004, 204-16).

More pragmatic reasons for embracing cryopreservation centered on resources and capacity. While the Jackson Lab and MRC Harwell maintained and supplied multiple strains, they could not keep up with all new variants. In 1976, Harwell used a quarter of its space purely to maintain stock and ensure the strains survived. With 120 mouse varieties, Mary Lyon (1976, 57), a prominent geneticist at Harwell, noted that they “carry only a small proportion of the several hundred mutant genes that are known in the mouse, since we cannot afford to devote any more space to stocks, and hence economy in stock-keeping could help us to extend our range of stocks, as well as being generally more efficient.”

Mouse genetics institutions and funders therefore embraced cryopreservation enthusiastically. The Jackson Lab started its embryonic archive in 1978 (Mobraaten 1981). The MRC sponsored Whittingham’s work as a core part of Anne McLaren’s Unit at University College, London, as well as frozen archives at Harwell and the Experimental Embryology and Teratology Unit at Carshalton.^4^ Integrating freezing into institutions that prioritized standardization also involved ascertaining that stored embryos would reliably reproduce what was being preserved. Beyond honing the techniques, early work sought to establish frozen embryos as normal by studying the effects of freezing on mutation rates. Thus, Lyon exposed frozen embryos to X-rays to examine whether freezing would protect embryos from accumulating mutations in the absence of DNA replication and repair during cell division. With Whittingham and Peter Glenister, she also tried to emulate long-term exposure to background radiation (Lyon, Whittingham, and Glenister 1977). Cryopreserved mice withstood radiation exposure well, suggesting they could be stored indefinitely while maintaining the genetic definition of the original strain.

To most observers, these radiation experiments allayed concerns about storage, but some saw it as another selling point of the freezing technology. In his foreword to the proceedings of the 1980 workshop on freezing embryos at Harwell, G. J. R. Hovell, the Secretary of the International Council for Laboratory Animal Science (ICLAS), wrote, “Embryo preservation should also embrace elements of quality control which is of additional importance when stocks are to be transferred and introduced to other laboratories. Genetic Monitoring is an aspect of such control which ICLAS is also currently sponsoring…but the recommendations are not as widely applied as is desirable so a new promotion campaign should be mounted” (Hovell 1981, xii). Genetic monitoring was a relatively novel concern, with proposals for testing strains’ genetic identity as part of accreditation schemes that ensured standardization of animal supply but had largely focused on pathogens. In the UK, a pilot scheme run by the MRC Laboratory Animal Centre (LAC) in Carshalton used mandibular anatomy measurements as a quick test to ascertain the strain, and its staff voiced suggestions to establish “elite nuclear colonies” with a high degree of monitoring, which could then provide breeding stock for others (Festing 1974). These efforts were, however, rather marginal in the 1970s. The MRC disbanded the LAC in 1982, and it was not until the 2000s that issues of genetic quality control received major attention, this time with freezing readily available.

In the 1970s and 1980s, the ability to freeze down embryos was welcome as a means to ensure their preservation by removing them from the vicissitudes of passing time. Arresting embryonic development to create “suspended life” thus provided a means to “extend the present” with respect to the existence of the strain alone, independently of the fate of existing living colonies of animals. Uncoupling the two offered a new, cryopolitical, strategy: if before strain survival required a decision to actively keep breeding the mice, now it was possible to bank previous investment and labor in the form of frozen life. Simultaneously, the freezer promised a deferral to otherwise inevitable questions about limits to capacity. Once it was clear that cryogenic life was unchanged yet viable, it provided an alternative to making colonies live so that the strain might live or letting it die with the animals.

The concern over the security of strains has parallels across Cold War science, agriculture, and conservation. Similar anxieties over loss, existential risk, and limited resources extended to the preservation of blood samples, seeds, rare breeds, and endangered species, among other objects (Radin 2017; Peres 2019; Chrulew 2017; cf. Sepkoski 2020). The concerns and imaginaries over each kind of object were, however, shaped by the specifics of the communities that sought to preserve it, infrastructures, and political and cultural interests. Thus, blood samples have been stored in collections but also in laboratory freezers, distributed across multiple sites and research agendas (Radin 2017). Seed banking has relied on international organizations and major institutions, such as the CGIAR and the flagship Arctic seed bank in Svalbard (Peres 2019). Yet, as Helen Curry has shown, as tensions between security of storage and potential reuse of seeds grew, with increasingly limited resources, more was vested in having backups of frozen seeds across multiple collections rather than robust infrastructures (Curry 2022). Mouse geneticists similarly built on resources available to them. While some, like Klein, envisioned international storage facilities, the mixed network of academic institutions and private suppliers meant several nationally significant bodies took control over storage efforts. Mouse geneticists relied on the established mixed moral economy of sharing and purchasing animals to distribute efforts across these sites. Furthermore, different kinds of pressures shaped mouse cryopreservation since the 1980s, as we go on to discuss—pressures associated with animal welfare and with ensuring genetic reproducibility.

### Responsibility: Welfare, Nonsentience, and Care for the Strain

As embryo cryopreservation was made reliable and routine in the 1980s, it was not explicitly framed in welfare terms, despite major contemporaneous changes in the governance of animal research. In the 1990s, however, as laboratory animal care gained greater prominence as a regulatory and ethical impetus, freezing acquired a further purpose: improving animal well-being. In the UK, the *Animals (Scientific Purposes) Act 1986* (ASPA) replaced the Victorian *Cruelty to Animals Act 1876* that had governed experimentation for over a century of dramatic scientific change. Designed as a compromise between scientific interests, moderate welfare campaigners, and the regulatory needs of the Home Office, ASPA revised the system of licensing for animal experiments, covered a broader range of procedures (including breeding), and empowered veterinarians, animal technicians, and selected lay voices (Kirk and Myelnikov 2022).

Frozen embryos were a marginal concern for the civil servants designing ASPA. While breeding laboratory animals became subject of regulation for the first time in 1986, as did fetal forms from halfway through gestation, cryopreservation remained largely outside of the Act.^5^ Indeed, the very exclusion of embryos and gametes from legislative protection gestures at their different normative and political status to living mice, as nonsentient entities. The mechanisms worked into ASPA, however, expanded the scope of expertise and participation beyond scientists. Acting through the refashioned and empowered Animal Procedures Committee (APC), moderate welfare voices had a significant input into the governance of animal research in Britain. Among other subjects, the APC drove the promotion of the 3Rs as the guiding principle for animal research: *reduction* of the numbers of animals used; *refinement* of tools and experimental design to minimize pain, suffering, or distress; and *replacement* of laboratory animals with nonsentient alternatives (Davies et al. 2018). While formulated by W. M. S. Russell and R. L. Burch in the late 1950s, the 3Rs remained marginal to the governance of animal research until the diversification of the voices that had regulatory impact in the 1990s (Kirk 2018).

In the 1997 review into the functioning of ASPA, the APC paid special attention to the problem of overbreeding, responding both to concerns raised by antivivisection groups and from within the animal research community (Coghlan 1997). “Surplus” animals, bred to maintain colonies but not used experimentally, challenged efforts to reduce numbers. The APC hoped that market forces would encourage efficiency and minimize overbreeding, but it additionally promoted cryopreservation as good practice to address the issue (APC 1998, 86; LASA 1998). In this way, cryopreservation became a means to enact the 3Rs: replacing living, breeding populations of animals with frozen embryos entered the regulatory framework that set boundaries, and the direction of travel for socially acceptable animal research.

By encouraging the suspension of strains not in use through freezing, APC offered a way for researchers to enact humane principles without necessarily reducing the numbers of animals available for active research projects. Indeed, cryopreservation offered a double advantage regarding the *reduction* of surplus mice: it minimized animals bred for research, while also avoiding the cost of maintaining breeding colonies—thus freeing up resources to care for fewer mice. Furthermore, cryopreservation was enrolled into enacting the 3Rs as a *refinement* during transport, particularly with genetically altered animals. Shipping frozen, non-sentient materials bypasses welfare concerns about transporting live animals, enabling less cumbersome regulation and cheaper transport, while offering a means to introduce strains into facilities with few biosecurity risks (Peres and Roe 2022).

Suspending the reproduction of the strain through freezing embryos was doubly advantageous. Firstly, cryopreservation provided a means to keep the strain without continual breeding, avoiding the production of animals purely for maintenance. Secondly, frozen embryos are not sentient and thus not understood to suffer in the same sense as fully developed animals. We suggest that this opens new forms of care that, rather than focusing on the experiences of individual animals, apply to the survival of the strain. Good animal care is an important feature of the civic epistemologies of British animal research (Friese, Nuyts, and Pardo-Guerra 2019), yet simultaneously the work of caring for research animals is “complex and contradictory”: namely, the animal care staff are often seeking to address experimental harms with their care-work and thus reduce suffering in their charges (Roe and Greenhough 2021). Cryopreservation raises new possibilities by separating the survival of the strain (at least when not in use) from the possibility of harm. This mode’s cryopolitical strategy is not to allow the strain to die while not allowing mice to live. In contrast with other examples of cryopreservation to prevent nonhuman extinction—frozen zoos and seed banks—the “freezing down” of strains to *avoid* the continual breeding of laboratory animals is intentional and cast as a positive outcome.

In contrast to the regulatory logic of animal research that focuses on individual animals as protected subjects, in cryopreserving, it is not the individual animal but the strain that is not being allowed to die. Indeed, the embryo and semen donor are culled after their reproductive substances are collected, since animal regulation prohibits reusing animals in experiments. A strain is a particular kind of a kinship system. As Kroløkke (2019) has argued, cryopreservation of companion animal DNA privileges the interspecies kinship between a pet and its owners in promoting and conceptualizing the market for this service, yet such a logic applies differently to laboratory mice. While interspecies kinship, framed in evolutionary terms, underpins the use of mice as models for human biology, it is the *intra*specific kinship between animals—as manifested through inbreeding and standardization—that matters here and shapes cryopreservation strategies.

The relationships between mice and humans in scientific establishments are primarily framed in terms of care rather than kinship. Caring practices, mainly performed by animal technicians with great tacit skill (Roe and Greenhough 2021), are also materialized in the infrastructures of the animal house, including caging, bedding, feed, and enrichments (Kirk 2016). Cryopreservation has joined the arsenal of material care and been integrated with the language and ambitions of ethical governance of animal experimentation. Bronwyn Parry has described mouse lines as collectively authored performative works, made and maintained by multiple scientists and technicians across locations and generations (Parry 2020). Cryopreservation, with its ability to arrest living processes and create forms of frozen life that are amenable to storage and dissemination, plays a significant role in that work.

### Stability: Genetic Integrity and Colony Management

Presently, cryopreservation of mice is a routine service offered by large repositories, institutional transgenic and embryology facilities, and commercial entities. Unlike services marketed to pet owners that focus on individual animals (Kroløkke 2019), its focus is the genetically defined collective that comprises the strain.^6^ A strain is considered securely preserved if 30-50 samples of sperm are frozen from three to five males or if 250-300 embryos have been stored at the two-cell stage.^7^ The number of strains and mutants created has also grown tremendously, while some (such as C57B Black 6) remain popular for many decades. Cryopreservation has become routinized and works in tandem with an upscaling in the production of mouse models.

As Gail Davies (2013a) argues, maintaining mice requires “careful management of their reproductive capacities toward replication and away from mutation” (p. 136). Keeping the purity of inbred lines and transgenic mutants with a defined genetic background is a “serious challenge” for animal facilities (Benavides et al. 2020, 140): “[i]t contradicts all principles of genetic diversity in nature to develop an inbred strain and try to *freeze* a homozygote genome over generations for a long time period” (Wedekind, Reifenberg, and Hedrich 2012, 623, added emphasis). Here, Wedekind and colleagues use the concept of freezing metaphorically to refer to the “holding still” of a strain’s genotype; yet it highlights how low temperatures are deployed to extend the present by preventing the “natural” tendency of the inbred mouse strain genome to mutate over time.

Cryopreservation, then, can offer insurance against genetic drift and contamination because it creates a pool of gametes or embryos “frozen” in time that can be a backup to which a strain can be restored if necessary. One early example of this mode was the genetic contamination of the 101/H inbred strain. In 1988, biochemical analysis showed that the mice at Harwell carried alleles uncharacteristic of the strain, likely from an unanticipated cross with C3H/HeH mice kept in the same room—either through human error or mice escaping their cages (Glenister, Whittingham, and Wood 1990). The problem was solved by reestablishing the colony from a set of embryos frozen in 1980. An unusual case at the time, it shows how archived embryos have been held in reserve against the inherent instability of strains as much as external factors.

In recent years, researchers have been advised to maintain genetic authenticity through colony management practices. One example is backcrossing, whereby a colony’s genetic background is “refreshed” by introducing mice from the strain’s colony of origin every few generations. Doing so increases the prevalence of the original genotypes and counteracts spontaneous genetic mutations expected to have accumulated within the colony. This is achieved, in effect, by introducing mice from the colony’s evolutionary past. The supplier stock used in backcrossing is itself kept as still as possible by using cryopreserved material. Processes such as the Jackson Laboratories’ Genetic Stability Program use cryopreservation to “slow genetic drift dramatically and prevent genetic contamination from permanently corrupting a strain in the future” (Taft, Davisson, and Wiles 2006, 653). When cryopreservation becomes part of everyday breeding, it enables the extension of the population’s evolutionary present by enabling crosses between individuals from colonies closer or further from the strain’s original genotype.

In this way, cryopreservation grew from a way to insure against loss to a means of avoiding changes to the genetic integrity of colonies over time. This entails a shift in the temporality of cryopreservation with respect to the making of novel strains: instead of waiting until a project is over, freezing can be done proactively, with a view to managing the risk of genetic variation over time. It should be noted that advocates for this more proactive form of cryopreservation tend to hail from the community of individuals already working with this technology, for instance, at Harwell and the Jackson Laboratory. As Ian, a senior cryorepository scientist, put it:

There’s always been a case to argue that the archiving elements to a project is at the end of the process, when in actual fact I think it should be at the front…so you protect what you’ve made when you’ve made it, so you actually have a date stamp on when you’ve actually generated [it], rather than…when you’ve finished your experiments and you want to clear some cage space or your license has run out, which is not unusual to get a panic phone call from somebody. So you’ve got ten years maybe of genetic drift that you could have captured at the beginning.

Such a stance suggests a different temporal orientation toward cryopreservation, where it figures as a much more routinized aspect of strain making and management, rather than a project closure task, or a means to enable strains to move. It becomes preemptive and can become normative, as a means for researchers to care about the animals, experiment, and the future reproducibility of the eventual results.

Cryopreservation also involves practices of management and maintenance work that are familiar to other kinds of biobanks, such as seed banks or *Drosophila* stock centers (Chacko 2019; Bangham 2019). The archiving process involves a degree of quality control which, coupled with the state of suspended animation, guarantees to users the authenticity of their strain. In turn, this has potentially significant implications for the outcome of projects that require significant investment of resources and time, as highlighted in the quote from an academic researcher called Kieran when reflecting on the possible consequences of using a mouse sourced from other academics:

So they say, “Yeah, here’s the mouse…it’s fine, that’s the same one, you know, we’ve been breeding it ever since” but…there might be ten, twenty animal technicians between then that work with it, you see, and then don’t really know what’s happened to it…you shouldn’t be that untrustworthy but you have to be because you’re putting several years of your research money on this one male they’ve sent you…that’s your career on the line.

Hence, in this mode, archived strains offer an alternative to the uncertainty inherent to using animals originating elsewhere. Given current concern about the reproducibility of animal-derived data (Lloyd et al. 2015; Baker 2016; Nelson et al. 2021), the cryopreserved embryo in repositories with good quality control practices becomes a means to manage anxieties about genetic drift and the contingencies of mouse breeding. Frozen embryos and gametes went beyond a promising means to overcome limits to capacity: the ability to decouple the existence of the strain from the continual reproduction of animals was repurposed as insurance against the vagaries of sexual reproduction on the one hand, and from unknown research and husbandry practices on the other.

As one reporter noted in *Lab Animal*, “to assure reproducibility scientists need mice to perform with the consistency of chemical reagents” (Zeldovich 2017, 256), but mice “have an intrinsic genetic drive to change, with mutations accumulating [and] compromising the reproducibility of experimental data over time and place” (Taft, Davisson, and Wiles 2006, 649), jeopardizing the orderly accumulation of mice and data for the future. Ultimately, then, cryopreserved embryos came to be enrolled creating a standard for a particular strain because their latency excludes them from the uncertainties of reproductive time.

### Extending the Present by Keeping Strains on Ice

Having set out three ways in which cryopreservation has featured in the landscape of mouse research, we now turn to the implications of being able to cryopreserve strains. We argue that the shift away from maintaining breeding colonies of living animals to preserving strains as frozen life has, ultimately, promised a means to overcome limits around capacity, social acceptability, and genetic integrity. In other words, it becomes possible to “extend the present” (Lemke, this volume) and enable the continuation of processes—be they strains or research programs—beyond what may have otherwise been possible.

Our account showcases the multiple ways cryopreservation has enabled the present to be extended by making reversibility possible, to undo possible losses by using frozen embryos or sperm to recover strains at the appropriate time. Freezing down strains has been, at different times, a practice of insurance, efficiency, or assurance; each with its set of dominant priorities, risks, and opportunities. The security mode is discernible at the earliest stage; as geneticists sought to avoid the loss of valuable strains while overcoming the material limits to capacity. Here, the ability to freeze strains opens up the possibility of science being resilient against total loss if animals were to die or funding to run out. In contrast, the responsibility mode is characterized by cryopreservation as an alternative to unnecessary breeding. It allowed scientists to keep strains without the associated economic and reputational costs of surplus mice, thus proposing an imaginary of good science that is both efficient and humane, where ethics and economics can align. Finally, in the assurance mode, cryopreserved embryos and sperm become a means to ensure the stability of the strain and avoid the inherent uncertainties of sourcing animals.

In delineating these modes of cryopreservation, we do not wish to suggest radical change. In each period we have discussed, practitioners made claims about context-specific priorities and values, but these have been layered over preexisting concerns. Cold War anxiety about risks and vulnerability did not dissipate, but emphasis has shifted toward minimizing waste, routine preservation, and best practices approach as a risk-management strategy. Yet, although cryopreservation appears malleable over time, we discern an underlying cryopolitical focus on managing frozen mice as resources by extending the present.

Finally, we wish to highlight how cryopreservation unsettles the relationship between care and the animals that are the objects of scientific research. Bangham (2019, 133) has emphasized the practices of care that go into maintaining Drosophila stocks, for which reliable freezing is unavailable, “to stabilize otherwise dynamic, breeding and potentially unruly living organisms.” With the advent of cryopreservation, mouse geneticists likewise caring for their strains, developed alternatives to prevent genetic decay and loss without managing breeding colonies. Hence, we suggest that cryopreservation can be understood as a form of care for and about strains rather than for the individual mice.^8^ Care is therefore also key as curation of the stocks, with careful records kept of the strain, genotype data, parents, and embryo stage.

In this sense, there are wider spheres of concern that are part of the drive to cryopreserve—relating to the success of research projects, preserving labor and assurance of quality. At the same time, and as shown especially in “Security: Embryo Freezing and the Survival of Strains” subsection, there is normative relevance to reducing unnecessary breeding. Therefore, the cryopolitics of mouse research makes visible how not letting live can be seen as a form of care and simultaneously another avenue of instrumentalization.

## Conclusion

How, then, do these different cryopolitical modes contribute to our understanding of cryopolitics on the one hand, and animal research on the other? Our analysis points to new kinds of cryopolitical arrangements that have developed in mouse genetics, shaped both by the practices and motivations for freezing among geneticists and by the historical context of animal research in the UK. The security mode has much in common with other studies familiar from cryopolitical literature: its Cold War concern with scarcity and catastrophe, its impetus to future-proof research, and its direct links with the worlds of agricultural cryopreservation. The responsibility and stability modes are, we propose, unique to the world of mouse genetics. Freezing was part of the moral management strategy in the 1990s UK, specific to the country’s late-twentieth-century regulatory arrangements and the cultural concerns over animal experimentation (Friese, Nuyts, and Pardo-Guerra 2019; Kirk and Myelnikov 2022). It also, however, shifted the usual cryopolitical logic. Radin and Kowal (2017) argue that “the temporal horizon of cryopolitical life means it is uncertain whether ‘not letting die’ is an alternative technique for ‘letting die’…or for ‘making live’” (p. 8). While not letting the strain die, the cryopolitical focus is also on *allowing not to live*, to reduce animal numbers and potential suffering, with the related motivation of cutting economic costs. Arresting animal reproduction through freezing enables animal research to persist more efficiently while allaying public concerns. Cryopreservation of laboratory animals is as much about “not letting live” as it is about “not letting die.”

The stability mode adds further dimensions to the function of cryobanks, which tie it most immediately to the long history of standardization as promise and tool in managing animal research (Rader 2004). Through its history, the success of mouse geneticists has relied on scaling up projects—from mass mutagen and carcinogen screens of the postwar years (Rader 2004, de Chadarevian 2006) to the growing production of transgenic animals since the 1980s, to major international collaboration of the genomic age, most notably the knock-out mouse project (KOMP) aiming to produce animals defective for each known gene (Davies 2013b). Freezing played a role in these scaling-up projects, not only by freeing up space in animal facilities but also by navigating new issues raised by scaling up that we discuss under responsibility and stability. At the practice level, the new calls for systematic freezing to standardize strains and protect them from genetic drift and human error has also helped underpin a new decentralized vision of international science, one in which “collaboration and trust are sought through quality assurance rather than through creating collective identities” (Davies 2013b, 427).

By transforming frozen embryo collections from projects of indefinite storage to routine mechanisms of quality assurance, cryobanks are envisioned to function more as “working collections” (Jardine, Kowal, and Bangham 2019). Making cryopreservation into a routine procedure, part of everyday management of strain breeding, is a good example of how a cryopolitical intervention can shape the politics of mouse research. If a strain can be frozen, then it follows to some advocates like Ian that this should be done at the beginning, to capture the “purest” expression of the strain, minimizing genetic drift. The technique has moved from one used to extend the life of the strain by avoiding change to one used to assure the identity of the strain.

Freezing embryos and gametes is a means of deferring the death of a particular ontological form of the laboratory mouse—the strain—and thus is an extension of the core value of standardizing laboratory animals. The strain is not allowed to die by not letting the mice live, as putting reproduction “on ice” avoids the need for continually breeding animals. Kroløkke (2019) uses the concept of “cryo-vitality”—the potential liveliness that may emerge in the future—to convey the production of hopeful value through freezing, but mouse cryobanks exemplify how the concept may have a different valence at the individual and the collective level. We’ve argued that mouse cryobanks emphasize the diminishing of said vitality as a virtue with regard to the experience of individual mice. At the level of the strain, however, the very ability to prevent change over time enables the extension of the strain’s productivity by slowing down the tendency for change.

The cryopolitics of mouse research hinges on this deferral of liveliness: freezing offers a way to store strains while reducing the numbers of mice used, and the resources required to support them. Proactively cryopreserving allows for the capture of the prodigious output of contemporary bioscience without unduly stoking political and ethical concerns. It overcomes epistemic and moral challenges of animal research in the present, enacting forms of good science that protect the future of mouse genetics. Exploring the cryopolitics of mouse freezing ultimately points to the strain—and indeed, mouse genetics itself—being the focal point of these modes of insurance, as articulated in the bodies of mice. The promise of strain cryopreservation could be seen as effectively encouraging the vitality of science and animal research beyond the issues which we’ve previously identified (genetic drift and breeding failure/loss) while reducing the costs and likelihood of change. This raises questions about the ways we characterize them: are these practices forms of care, new frontiers of control and instrumentalization, both, or neither? We suggest that cryopreservation opens up new horizons for care and for instrumentalization by aligning and entangling the experiences of laboratory animals with the proper maintenance of a mouse strain. Doing so recognizes the welfare deficits associated with life in the laboratory, and it also reinforces the framing of mice as research tools whose genotype can be stabilized through technology. The advantage of bringing together social scientific and historical perspectives to bear on this account is that it demonstrates that the answer must be historically specific, evolving, and ultimately informed by the broader social and historical context of cryopreservation.

## Notes

1. By contrast with human reproductive medicine, mouse oocytes are not routinely frozen to preserve strains, even though techniques for oocyte freezing have existed since the 1970s. Semen is easier to store, and the ability to induce mice to breed means embryos can be easily produced, reducing the need for performing in vitro fertilization (Glenister and Thornton 2000).

2. Data were collected as part of the Animal Research Nexus (www.animalresearchnexus.org), Histories and Cultures and the Markets and Materials subprojects. Interview and ethnographic data were thematically coded using NVivo. Ethnographies and interview recruitment happened in accordance with ethical approval from the University of Southampton (ERGO nr 30875).

3. The genetic definition is that an inbred strain is essentially homozygous at all loci (Mouse Genome Informatics, http://www.informatics.jax.org/glossary/inbred_strain, accessed on March 15, 2022), which corresponds to the genetic concept of a pure line.

4. The National Archives, UK (TNA): FD 12/1199, “MRC Mammalian Development Unit…” April 1984.

5. TNA: HO 285/189, “Discussion of Policy…” April 27, 1984.

6. Infrafrontier: the European Mouse Mutant Archive (https://www.infrafrontier.eu/infrafrontier-research-infrastructure/organisation/european-mouse-mutant-archive-%E2%80%93-emma, accessed September 8, 2022).

7. MRC Harwell Archive FAQ (https://www.har.mrc.ac.uk/wp-content/uploads/FAQs-2021.pdf, accessed April 14, 2022).

8. There are complexities in drawing this distinction, for example, having fewer strains as colonies means that those existing animals can have more attention and resources for care, but it broadly holds.
